# *Dendrobium huoshanense* Ameliorates Sleep Deprivation-Induced Ileal Mucus Barrier Dysfunction by Regulating Steroid Hormone Biosynthesis and the HPA Axis in Rats

**DOI:** 10.3390/metabo16060376

**Published:** 2026-05-30

**Authors:** Xue Luo, Shuxiang Jin, Yue Fang, Qun Zhao, Huiqun Xie, Lan Han

**Affiliations:** 1 School of Pharmacy, Anhui University of Chinese Medicine, Hefei 230011, China; 2023205225068@stu.ahtcm.edu.cn (X.L.); 2023205225073@stu.ahtcm.edu.cn (S.J.); rscszk@ahtcm.edu.cn (Y.F.); 2 College of Life and Health, West Anhui University, Lu’an 237012, China; 3 MOE-Anhui Joint Collaborative Innovation Center for Quality Improvement of Anhui Genuine Chinese Medicinal Materials, Hefei 230012, China; 4 Institute of Traditional Chinese Medicine Resources Protection and Development, Anhui Academy of Chinese Medicine, Hefei 230012, China

**Keywords:** *Dendrobium huoshanense*, intestinal mucosal barrier, sleep deprivation, UPLC-QTOF-MS

## Abstract

**Background/Objectives**: Sleep deprivation (SD) induces the accumulation of reactive oxygen species (ROS) in the intestine, causing inflammation in the intestine, thereby damaging the intestinal epithelial barrier function. As a traditional Chinese medicine, *Dendrobium huoshanense* (DHS) modulates intestinal flora, maintains the intestinal mucosal barrier, and promotes gastrointestinal motility and digestive secretion. However, the role and mechanism of DHS in improving SD-induced intestinal injury have not been fully studied. **Methods**: The SD model was established by subjecting rats to complete SD using a specialised SD instrument. Hematoxylin and eosin (HE) staining was performed to evaluate pathological injury in ileal tissues. Enzyme-linked immunosorbent assay (ELISA) and biochemical methods were used to quantify the main inflammatory cytokines, oxidative stress markers, and hypothalamic–pituitary–adrenal (HPA) axis activity. The expression levels of E-cadherin and Occludin proteins in the ileum tissue were analyzed by Western blotting. Additionally, the pH value of ileal mucus, unit secretion, water content, and dry matter weight were measured. Differential metabolites in rat ileum mucus were profiled using ultra-high-performance liquid chromatography–quadrupole time-of-flight mass spectrometry (UPLC-QTOF-MS). **Results**: DHS alleviated the pathological injury of the ileum induced by SD. DHS reduced the levels of serotonin (5-HT), interleukin-6 (IL-6), and tumor necrosis factor-α (TNF-α), while increasing interleukin-10 (IL-10) levels, thereby attenuating systemic inflammatory responses. Furthermore, DHS decreased malondialdehyde (MDA) content and elevated glutathione (GSH) and superoxide dismutase (SOD) levels in ileal tissues. DHS also upregulated the protein expression of E-cadherin and Occludin in intestinal tissues. In addition, DHS decreased the pH of ileal mucus, promoted intestinal mucus secretion, and increased dry matter content, facilitating the restoration of the mucus barrier. DHS may alleviate SD-induced ileal injury by modulating steroid hormone biosynthesis. DHS decreased the levels of adrenocorticotropic hormone (ACTH), cortisol (CORT), and corticotropin-releasing hormone (CRH), indicating that DHS suppresses the abnormal activation of the hypothalamic–pituitary–adrenal (HPA) axis. **Conclusions**: In this study, a comprehensive multi-index evaluation showed that DHS could significantly improve the ileal injury caused by SD in rats. The mechanism involved regulating the balance of serum neurotransmitters and inflammatory factors, reducing oxidative stress in tissues, and improving the physicochemical properties of intestinal mucus. Metabolomic analysis further revealed that these protective effects may be mediated via the regulation of steroid hormone biosynthesis pathways and are associated with the inhibition of abnormal HPA axis activation.

## 1. Introduction

Sufficient and high-quality sleep is fundamental to maintaining normal physiological functions in the human body. However, amid the accelerated pace of modern life, insufficient sleep has emerged as a widespread public health concern [1]. Epidemiological and clinical evidence consistently demonstrates that chronic sleep disturbances are closely associated with elevated risks of numerous chronic disorders, including immune dysfunction, oxidative stress injury, endocrine disorders, and neurological deficits, among other physiological and pathological alterations [2,3]. In recent years, the bidirectional interaction between sleep and gastrointestinal health, known as the “gut–brain axis,” has attracted substantial research attention [4]. The gut–brain axis (GBA) functions as a pivotal network connecting gut microbiota with the central nervous system, where sleep deprivation (SD) disrupts gut motility, mucosal integrity, and microbial composition, while microbial metabolites in turn regulate neurotransmission and inflammatory signaling [5].

The intestine is not only an essential organ for digestion and absorption but also a dynamic ecosystem exerting complex neuroendocrine and immune functions. Its homeostasis is critical for systemic health [6]. SD can directly impair intestinal barrier function, thereby triggering intestinal inflammation and oxidative stress. Specifically, SD may lead to increased intestinal permeability, downregulation of tight junction protein expression, and excessive accumulation of reactive oxygen species (ROS) [7,8]. Recent studies have discovered that SD specifically targets intestinal stem cells via the vagus nerve-dorsal motor nucleus nerve (DMV)-5-hydroxytryptamine (5-HT) axis, causing ROS overproduction and impairing intestinal barrier repair [9]; the research revealed that SD-induced intestinal ROS accumulation can also be mediated by d-serine-NMDAR signaling and mitochondrial metabolic disorders [10]. Notably, SD-induced gut damage also involves ferroptosis, a lipid peroxidation-dependent cell death pathway regulated by ALOX15 signaling, which precedes lethal ROS accumulation and specifically damages goblet cells [11]. These findings collectively indicate that SD-induced intestinal oxidative stress is a multi-pathway mediated pathological process, and targeted intervention of this process holds great promise for preventing SD-related gastrointestinal diseases.

*Dendrobium huoshanense* (DHS) is a rare medicinal orchid species endemic to China. Modern pharmacological studies have demonstrated that DHS is abundant in polysaccharides, alkaloids, amino acids, and diverse trace elements, and exhibits potent antioxidant, anti-inflammatory, immunomodulatory, and neuroprotective activities [12]. Specifically, *Dendrobium huoshanense* polysaccharides (DHP) have been shown to upregulate the Nrf2/HO-1 antioxidant pathway, reduce ROS and malondialdehyde (MDA) levels, and elevate superoxide dismutase (SOD) activity in animal models [13]. Multiple studies have documented its protective effects against chemically induced or inflammatory intestinal injury. Isovitexin isolated from DHS attenuates ulcerative colitis by repairing the intestinal epithelial barrier and modulating gut microbiota [14]. Accumulating evidence supports its ability to maintain intestinal homeostasis against chemical or inflammatory intestinal insults [15,16]. The DHP modulates the abundance of beneficial bacteria such as Bifidobacterium and Faecalibaculum, and regulates short-chain fatty acid (SCFA) metabolism to maintain intestinal homeostasis [17]. Given that SCFAs have been shown to ameliorate TNF-α-induced intestinal oxidative stress by upregulating antioxidant enzyme activities [17], the prebiotic-like effects of DHS further support its potential in intestinal protection. Despite the well-documented antioxidant and intestinal protective effects of DHS, there is a critical research gap: no study has explored its regulatory role in SD-induced intestinal injury.

On this basis, we hypothesize that DHS may exert a protective effect against SD-induced ileal injury by attenuating inflammatory responses and oxidative stress, restoring the intestinal epithelial mucosal barrier, and modulating intestinal mucosa-related metabolic pathways.

## 2. Materials and Methods

### 2.1. Study on the Basic Pharmacodynamics of DHS on Sleep-Deprived Rats

#### 2.1.1. Dendrobium Huoshanense Water Extract

DHS was purchased from Jiuxianzun *Dendrobium huoshanense* Co., Ltd. (Lu’an, China). The dried stems of DHS were ground into a fine powder using a pulverizer and then subsequently sieved through a No. 6 sieve to obtain the dried powder. Forty grams of the dried DHS powder was weighed, and a 70-fold volume of distilled water was added. The mixture was subjected to reflux extraction in a water bath at 100 °C for 1.5 h. After each water bath extraction, the extract was vacuum filtered, and the filter residue was extracted by water bath again until the last extraction. This extraction procedure was repeated three times. The resulting aqueous extracts from the three reflux extractions were combined, concentrated to 143 mL using a rotary evaporator, and prepared as an aqueous extract solution equivalent to 0.28 g·mL^−1^ of the raw medicinal material. (calculated based on the mass of the original medicinal material divided by the volume of the final concentrated liquid). Diluted with distilled water to the required middle- (0.14 g/mL) and low- (0.07 g/mL) dose concentrations.

#### 2.1.2. Animal Modeling and Dosage Regimen

The experimental protocol was approved by the Animal Ethics Committee of Anhui University of Traditional Chinese Medicine (approval code: AHUCM-rats-2023185). The Sprague Dawley rats used in the experiment were placed in a controlled environment (22 ± 2 °C, 50 ± 5% relative humidity, 12 h light and dark cycle), freely fed with standard food and water adaptive feeding for 1 week.

In this study, 60 Sprague Dawley rats were divided into two batches for experiments. Sleep deprivation instrument (Calvin, Nanjing, China) of a transparent plexiglass cavity (diameter: 36 cm, length: 40 cm) with a linear circulating stirrer at the bottom that physically interferes with the rat to prevent them from falling asleep. with automatic random clockwise/counterclockwise rotation at 20 rpm, equipped with a corn cobbed base to avoid animal injury. Among them, 36 rats were used to study the basic pharmacodynamics of DHS on sleep-deprived rats. After 3 days of adaptive feeding in the sleep deprivation instrument, they were randomly divided into 6 groups according to body weight, 6 rats in each group: control group (Con), model group (Mod), positive drug group (vitamin E), high-dose DHS group (DHS-H), middle-dose DHS group (DHS-M), and low-dose DHS group (DHS-L).

Twenty-four rats were subjected to metabolomics analysis of ileum mucus by DHS. After 3 days of adaptive feeding in a sleep deprivation instrument, the rats were randomly divided into 3 groups according to body weight, with 8 rats in each group: control group (Con), model group (Mod), and high-dose DHS group (DHS-H).

Different doses of DHS water extract (0.7, 1.4, 2.8 g/kg/d) and vitamin E (50 mg/kg/d) were intragastrically administered at 9:00 in the morning, and SD was performed at 13:00 in the afternoon for 24 h for 3 consecutive days. The rats in the control group were placed in the same device for free sleep, and distilled water was administered daily according to the body weight of the rats at a dose of 1 mL/100 g. At the end of the modeling administration, urethane was used to anesthetize the rats. After confirming the anesthesia of the rats, blood was taken from the abdominal aorta to obtain serum. The ileal segment was removed from the beginning of the branch of the mesenteric aortic arch to the end of the junction with the cecal segment.

#### 2.1.3. Histopathological Examination of the Ileum

The ileum tissue was fixed in 4% paraformaldehyde fixative for 24 h. The tissue was dehydrated using a graded ethanol series, cleared with xylene, embedded in paraffin, and serially sectioned into 4-µm-thick sections (RM2016, Leica, Wetzlar, Germany) for hematoxylin and eosin (H&E) staining. Pathological changes in the ileum tissue were observed under an optical microscope (CX41, Olympus, Hachioji, Japan), and digital images were acquired.

#### 2.1.4. Detection of Serum Inflammatory Factors

Serum levels of 5-HT, IL-6, IL-10, TNF-α, ACTH, CORT, and CRH were measured according to the instructions provided in the assay kits (Jianglai, Shanghai, China). All kits were balanced to room temperature before use. The serum samples were appropriately diluted and added to a pre-coated 96-well plate, and then incubated at 37 °C for 60 min. After washing the plate repeatedly for 3 times, the second antibody was added and incubated at 37 °C for 30 min. After the plate was washed three times again, the TMB substrate solution was used to color in the dark at 37 °C for 15 min, and the reaction was terminated with the termination buffer. The absorbance was measured at 450 nm using a full-wavelength microplate reader. Standard curves were constructed with standard concentration on the x-axis and absorbance on the y-axis. The concentrations of 5-HT, IL-6, IL-10, TNF-α, ACTH, CORT, and CRH were then calculated using the respective standard curve equations.

#### 2.1.5. Detection of Oxidative Stress Level in the Ileum Tissue

The rat ileum tissue was taken out from the refrigerator at −80 °C, and 0.1 g was weighed in a 1.5 mL EP tube, and 1 mL normal saline was added. An appropriate amount of small steel balls was added to each tube and homogenized on a tissue homogenizer. After homogenization, it was centrifuged at 4 °C, 4000 g/min, and centrifuged for 15 min. The separated supernatant was taken and stored in a refrigerator at −80 °C. The total protein concentration in the homogenate supernatant was determined using the BCA kit. GSH, MDA content, and SOD activity were calculated according to the kit instructions and formulas (Jiancheng, Nanjing, China).

(1) Malondialdehyde (MDA) content: MDA was detected by the Thiobarbituric Acid (TBA) method. MDA was condensed with TBA to form a red product with a maximum absorption peak at 532 nm.

$$\begin{array}{c} MDA\\ \left(nmol/mg\right) \end{array}=\frac{{OD}_{\left(sample\right)}-{OD}_{\left(control\right)}}{{OD}_{\left(standard\right)}-{OD}_{\left(blank\right)}}\times C_{\left(standard\right)}÷Total\;protein\;content$$

where $C_{\left(standard\right)}=10\;nmol/mL$.

(2) Superoxide dismutase (SOD) activity: Superoxide dismutase (SOD) enzymatic activity was determined using the WST-1 method. The xanthine–xanthine oxidase system was employed to continuously generate superoxide anion radicals in the reaction solution. WST-1 reagent can be reduced by superoxide anions to produce orange formazan compounds, which have a maximum absorption peak at 450 nm. SOD activity was calculated by detecting its inhibitory ability on the production of formazan.

$$\begin{array}{c} SOD\;activity\\ \left(U/gprot\right) \end{array}=\frac{{OD}_{\left(control\right)}-{OD}_{\left(sample\right)}}{{OD}_{\left(control\right)}}÷50\%\times Dilution\;factor÷Total\;protein\;content$$

(3) Glutathione (GSH) content: GSH can react with dithiodinitrobenzoic acid (DTNB) to form a yellow compound, which can be detected at 405.

$$\begin{array}{c} GSH\;content\\ \left(\mu mol/prot\right) \end{array}=\frac{{OD}_{\left(sample\right)}-{OD}_{\left(blank\right)}}{{OD}_{\left(standard\right)}-{OD}_{\left(blank\right)}}÷C_{\left(standand\right)}\times 2÷Total\;protein\;content$$

where $C_{\left(standard\right)}=20\;\mu mol/L$.

#### 2.1.6. Detection of Tight Junction Protein in the Ileum Tissue

Fifty milligrams of ileal tissue was weighed and mixed with protein lysis buffer containing protease inhibitor (Labgic, Beijing, China) and phosphatase inhibitor (Labgic, Beijing, China). The mixture was thoroughly homogenized at 4 °C using a tissue homogenizer operating at 70 Hz with 50 s of homogenization followed by 10 s of cooling, repeated for 6 cycles. Subsequently, the homogenate was incubated on ice for an additional 30 min to facilitate protein release. After pre-cooling the centrifuge to 4 °C, the homogenate was centrifuged at 12,000 r·min^−1^ for 20 min, and the supernatant was collected. Protein concentration was determined using the BCA assay (Labgic, Beijing, China), and then 5 µg of protein per well was loaded for electrophoresis on a 10% SDS-PAGE gel (Labgic, Beijing, China). After electrophoresis, the separated proteins were transferred onto a PVDF membrane (0.45 μm pore size). The membrane was blocked with 5% skim milk on a shaker for 1 h and then incubated overnight at 4 °C with primary antibodies against Occludin (1:1000), E-cadherin (1:3000), and GAPDH (1:3000) (Proteintech, Wuhan, China). The membrane was then incubated with a secondary antibody (1:20,000) (Biodragon, Beijing, China) on a shaker at room temperature for 2 h. Protein bands were visualized using an ECL chemiluminescence substrate, and band intensities were quantified using ImageJ 1.54g (National Institutes of Health, Bethesda, MD, USA).

#### 2.1.7. Physicochemical Properties of Ileal Mucus

Following euthanasia, the ileal segments were collected. Each ileal segment was opened longitudinally along the intestinal lumen. Solid contents within the segments were carefully removed using forceps until the mucosal surface appeared visually clean. Intestinal mucus samples were collected by gently scraping the mucosal surface with a smooth glass slide and were then transferred into 1.5 mL centrifuge tubes. The pH of the mucus was immediately measured using a pH meter (Leici, Shanghai, China), after which the samples were rapidly frozen in liquid nitrogen.

The weight of an empty centrifuge tube (M1) was recorded in advance using a precision electronic balance. After mucus collection, the sample was placed in the pre-weighed centrifuge tube, and the total weight (M2) of the intestinal mucus from each segment was measured. The length of each ileal segment (L) was also recorded. The unit secretion rate of intestinal mucus per segment (C) was calculated using the corresponding formula.

$$C=\frac{M2-M1}{L}\cdot (g\cdot {cm}^{-1})$$

The collected intestinal mucus samples from each segment were thoroughly frozen at −80 °C and then transferred to a freeze-dryer (Jinshi, Nanjing, China). The cold trap temperature was set to −80 °C, and the vacuum level was maintained at −3.1 kPa during 24 h of freeze-drying. After the intestinal mucus was completely dried, its weight (M3) was recorded. The water content (W1) and dry matter content (W2) of the intestinal mucus in each segment were calculated using the corresponding formulas.

$$W1=\frac{\left(M2-M1-M3\right)}{\left(M2-M1\right)}(\%)$$

$$W2=\frac{\left(M3-M1\right)}{\left(M2-M1\right)}\cdot (g)$$

### 2.2. Metabolomic Analysis of Ileal Mucus

#### 2.2.1. Preparation of Ileal Mucus Samples

The rat ileal mucus samples were removed from −80 °C and thawed on ice. Exactly 100 mg of the mucus was weighed into a 1.5 mL centrifuge tube. Then, 600 μL of pre-cooled methanol containing 25 μg·mL^−1^ 2-chloro-L-phenylalanine (internal standard) (Macklin, Shanghai, China) was added. The mixture was vortexed for 30 s. Two small steel beads were added, and the sample was thoroughly homogenized at 4 °C using a tissue homogenizer operating at 70 Hz with 10 s of homogenization followed by 10 s of cooling, repeated for 9 cycles. This was followed by low-temperature ultrasonication for 1 h, vortexing for 1 min, a second round of low-temperature ultrasonication for 1 h, and final vortex mixing. The mixture was then centrifuged at 4 °C and 12,000 r·min^−1^ for 15 min. After centrifugation, 500 μL of the supernatant was transferred to a new tube. From this 500 μL aliquot, 40 μL was taken from each sample and pooled to prepare QC samples. Finally, the samples were dried using a vacuum centrifugal concentrator (1500 rpm, 24 h, 4 °C) (Jiaimu, Beijing, China), sealed, and stored at −80 °C. Before analysis, the samples were reconstituted in 100 μL of methanol, filtered through a 0.22 μm membrane, and then subjected to instrumental analysis.

#### 2.2.2. UPLC-Q-TOFMS Analysis Conditions

Liquid chromatography was performed using an Agilent 1290 liquid chromatography system (1290-6546, Agilent, Santa Clara, CA, USA), with the column temperature maintained at 40 °C. Metabolites were separated on an Xselect^®^ HSS T3 column (4.5 × 150 mm, 2.5 μm) (Waters, Milford, MA, USA). Mobile phase A consisted of water containing 0.1% formic acid, and mobile phase B was methanol. A gradient elution program was applied as follows: 0–6 min, 10% B → 30% B; 6–12 min, 30% B → 80% B; 12–32 min, 80% B → 100% B; 32–35 min, 100% B. This was followed by a 6-min post-run equilibration period. The flow rate was 0.5 mL·min^−1^, and the injection volume was 2 μL.

Mass spectrometry was performed using an Agilent 6546 Q-TOF-MS system (quadrupole time-of-flight mass spectrometer) operating in both positive and negative ion modes. The precursor ion scan range was set to *m*/*z* 20–1000. Other key detection parameters were set as follows: capillary voltage: 4000; drying gas flow: 8 L·min^−1^; nebulizer pressure: 35 psig; gas temperature: 325 °C; sheath gas flow: 11 L·min^−1^; sheath gas temperature: 350 °C.

#### 2.2.3. Ileal Metabolomics Data Analysis

All UPLC-TOF-MS data were preprocessed using MassHunter Profinder software15.0 (MPP15.0) for peak alignment and integration, generating a three-dimensional data matrix comprising retention time, mass-to-charge ratio, and peak intensity. The resulting three-dimensional data matrix was imported into the metabolomics data analysis platform MetaboAnalyst 6.0 (https://www.metaboanalyst.ca (accessed on 20 March 2026)) for quality control (RSD < 20%), missing value imputation, and normalization. Differential metabolites that were statistically significant between the Con and Mod groups, as well as between the Mod and DHS-H groups, were screened using the following criteria: FDR < 0.05 and VIP > 1, combined with either FC > 2 (for upregulated metabolites) or FC < 0.5 (for downregulated metabolites).

The processed data were then imported into Mass Profiler Professional 15.0 (MPP15.0) software and matched against the METLIN database. Combined with the Human Metabolome Database (HMDB) and the Kyoto Encyclopedia of Genes and Genomes (KEGG), differential metabolites were identified.

The identified differential metabolites were imported into MetaboAnalyst 6.0 for KEGG-based metabolic pathway enrichment analysis. Metabolic pathways with *p* < 0.1 and pathway impact > 0.0 were considered significant. This analysis aimed to elucidate the mechanism of action of DHS-H in sleep-deprived rats.

### 2.3. Statistical Analysis of Data

All data were expressed as mean ± standard deviation (SD). Statistical analysis was performed using GraphPad Prism 10 (GraphPad Software, San Diego, CA, USA). One-way analysis of variance (ANOVA) followed by Tukey’s post hoc test was used for multiple comparisons. Differences were considered statistically significant at *p* < 0.05, *p* < 0.01, and *p* < 0.001.

## 3. Results

### 3.1. Sleep Deprivation Leads to Pathological Damage of the Ileum Tissue in Rats

**Figure 1:**
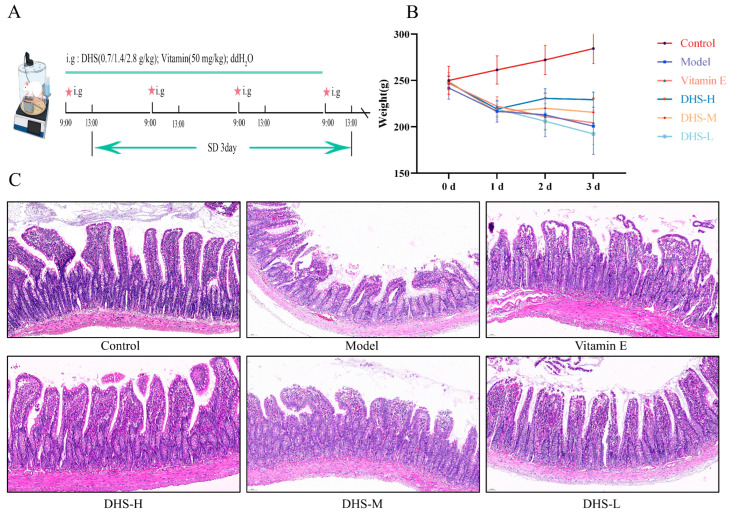
Sleep deprivation leads to weight loss and ileal pathological damage in rats. (**A**) Schematic of the experimental dosing schedule. (**B**) Body weight of rats in each group. (**C**) Pathological sections of the ileum tissue of rats in each group (50 μm) (mean ± SD, *n* = 6).

During the experimental period, the rats in the Con group were in good mental state, with smooth and shiny fur and steady weight gain. In contrast, rats in the Mod group showed hyperactivity on the first day of modeling, accompanied by a significant decrease in body weight. As SD continued, by the third day, these rats displayed dull and shedding fur, a lethargic state, reduced activity, and almost no desire to struggle or resist. Compared with the Con group, the body weight of the Mod group decreased continuously during the modeling period. As illustrated in Figure 1B, compared with the Mod group, rats treated with either high- or medium-dose DHS (DHS-H and DHS-M, respectively) exhibited only a transient reduction in body weight on the first day of modeling, followed by a steady and gradual increase over the subsequent 48 h.

As shown in Figure 1C, the results of HE staining showed that compared with the Con group, the ileum tissue of the Mod group was slightly edematous, the intestinal villi were broken, the villus epithelial tissue was fragmented, the arrangement was disordered, and the crypt was dilated. After administration, the intestinal villus crypts were arranged in an orderly manner, and the villus length increased. After treatment, the edema of villi was reduced, the fragmentation was reduced, the structure was arranged neatly, and the damage state was significantly improved.

### 3.2. DHS Can Alleviate the Inflammatory Response Caused by Sleep Deprivation

**Figure 2:**
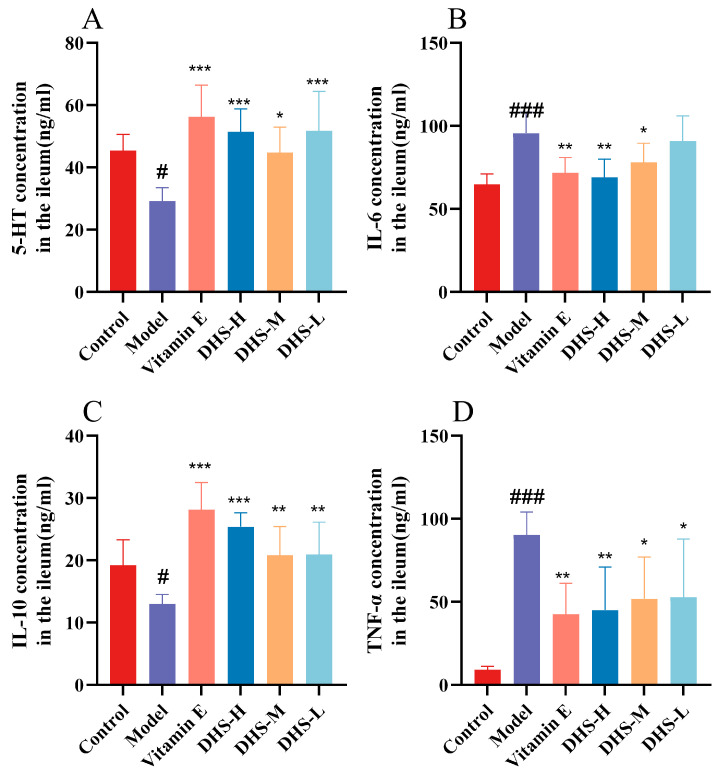
Serum markers showed that DHS could alleviate the inflammatory response caused by sleep deprivation. (**A**–**D**). The effects of each group on 5-HT, IL-6, IL-10, and TNF-α in serum (mean ± SD, *n* = 6). Compared with Con group #, *p* < 0.05; ##, *p* < 0.01; ###, *p* < 0.001; compared with Mod group, *, *p* < 0.05; **, *p* < 0.01; ***, *p* < 0.001.

The SD rat model was evaluated by measuring serum levels of 5-HT. As a key neurotransmitter in the gut–brain axis, 5-HT is synthesized and secreted by enterochromaffin cells and plays an important role in the sleep-wake cycle, particularly in promoting wakefulness and suppressing REM sleep. As shown in Figure 2, after 3 days of SD, 5-HT levels in the Mod group were significantly reduced, while the Vitamin E group and all DHS groups showed markedly elevated levels compared to the Mod group. Compared to the Con group, SD led to a significant decrease in the anti-inflammatory factor IL-10 and a notable increase in the pro-inflammatory factors IL-6 and TNF-α. Administration of Vitamin E and DHS significantly attenuated the SD-induced abnormal changes in cytokine levels.

### 3.3. DHS Can Reverse the Damage to the Ileal Intestinal Barrier Caused by Sleep Deprivation

**Figure 3:**
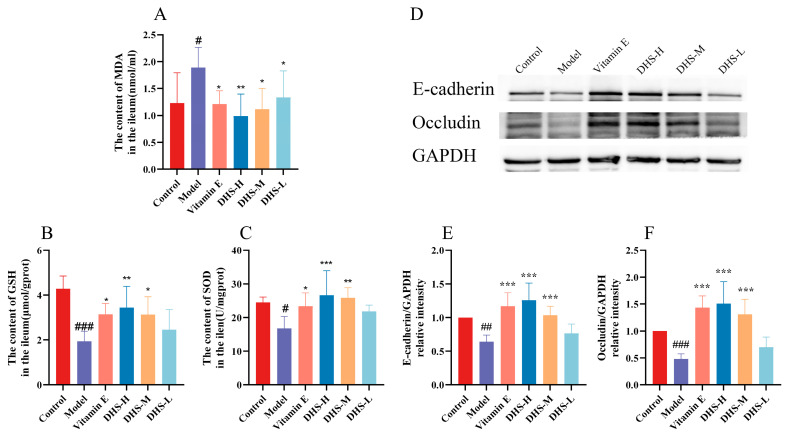
Ileal biochemical markers and Western blotting showed the effect of DHS on the damage of the ileal intestinal barrier caused by sleep deprivation. (**A**) MDA content; (**B**) GSH content; (**C**) SOD activity. (**D**) The expression of E-cadherin and Occludin protein in the ileum tissue. GAPDH was used as the protein loading control. (**E**) Density measurement of Occludin expression. (**F**) Density measurement of E-cadherin expression (mean ± SD, *n* = 6). Compared with Con group #, *p* < 0.05; ##, *p* < 0.01; ###, *p* < 0.001; compared with Mod group, *, *p* < 0.05; **, *p* < 0.01; ***, *p* < 0.001.

SD can lead to the accumulation of ROS in the intestine, and ROS acts as an oxidant. Excessive amounts can trigger damage such as lipid peroxidation and produce markers such as MDA. The cellular antioxidant system maintains redox balance through enzymatic defenses (such as SOD scavenging superoxide anions) and non-enzymatic defenses such as GSH scavenging ROS directly or as a glutathione peroxidase substrate) to prevent damage [7,18]. As shown in Figure 3A–C, compared with the Con group, the Mod group showed a significant increase in MDA content and significant decreases in GSH and SOD levels. After administration of Vitamin E and DHS, MDA content was significantly reduced, while GSH and SOD levels increased. These results indicate that DHS can effectively alleviate the intestinal oxidative stress state induced by SD.

The expression level of tight junction protein in the ileum was detected by Western blot. As shown in Figure 3D–F, compared with the Con group, the expression of E-cadherin and Occludin protein in sleep-deprived rats was significantly decreased. On the contrary, supplementation of vitamin E and DHS attenuated the stimulation of SD on these two tight junction proteins in the ileum of rats, resulting in a significant increase in the expression levels of these two tight junction proteins.

### 3.4. DHS Can Restore the Ileum Mucus Balance Caused by Sleep Deprivation

**Figure 4:**
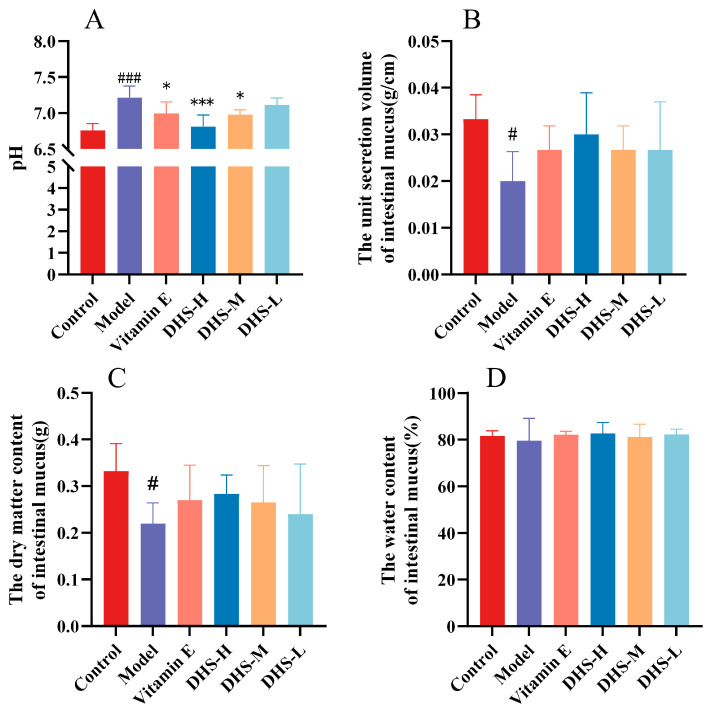
Effects of DHS on ileal mucus balance induced by sleep deprivation. (**A**) pH value of ileal mucus. (**B**) The Unit secretion volume of intestinal mucus (g/cm); (**C**) The dry matter content of intestinal mucus (g). (**D**) The water content of Intestinal mucus (%) (mean ± SD, *n* = 6). Compared with Con group #, *p* < 0.05; ##, *p* < 0.01; ###, *p* < 0.001; compared with Mod group, *, *p* < 0.05; **, *p* < 0.01; ***, *p* < 0.001.

pH value is one of the factors influencing mucus formation and release, as it can regulate mucus secretion [19]. Changes in the thickness and chemical composition of the intestinal mucus layer and increased penetration of the bacterial mucus barrier can be used to respond to the state of the gut [20,21]. As shown in Figure 4, compared with the Con group, the pH value of ileal mucus in the Mod group was significantly elevated. After administration, the pH values in all treatment groups showed varying degrees of recovery. The unit secretion rate of ileal mucus was also measured. Compared with the Con group, the Mod group exhibited a reduction in the unit secretion rate of ileal mucus, along with a decrease in its dry matter content. In comparison with the Mod group, all treatment groups demonstrated varying degrees of improvement in both mucus secretion rate and dry matter content, although the differences were not statistically significant.

### 3.5. DHS Improved Ileal Mucus Metabolomics Analysis in Sleep Deprivation Rats

**Figure 5:**
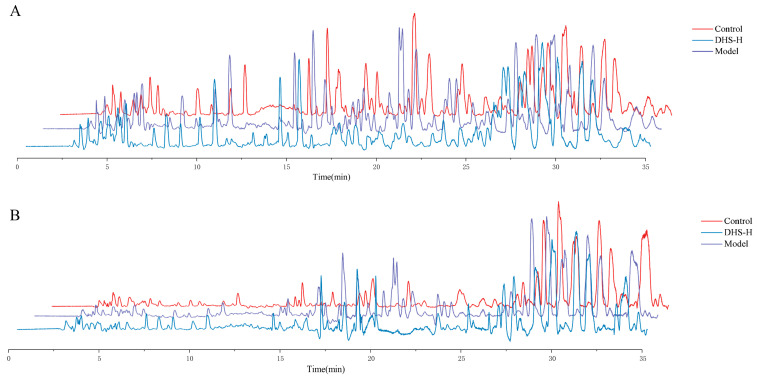
UPLC-Q-TOF-MS total ion chromatogram. (**A**) Con, Mod, and DHS-H total ion chromatograms in positive ion mode; (**B**) Con, Mod, and DHS-H chromatograms in negative ion mode.

Ileal mucus samples were analyzed using UPLC-Q-TOF-MS in both positive ion mode (ESI^+^) and negative ion mode (ESI^−^). Representative total ion chromatograms (TICs) of ileal mucus for each group under positive and negative ion modes are shown in Figure 5A,B. In the positive and negative ion modes, there were differences in the metabolic profiles of intestinal mucus in each group. The number and integrated area of several chromatographic peaks differed significantly among groups, and the peaks were generally well separated.

**Figure 6:**
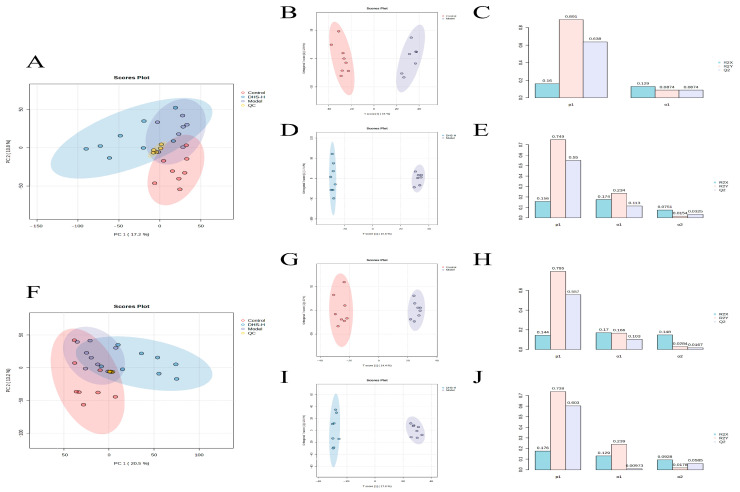
Multivariate statistical results of the intestinal mucus sample group. (**A**–**E**) In positive ion mode: (**A**) principal component analysis; (**B**) OPLS-DA score plot of Con compared with Mod group; (**C**) OPLS-DA verification diagram; (**D**) OPLS-DA score plot of Mod compared with DHS-H group; (**E**) OPLS-DA verification diagram; (n = 7–8). (**F**,**G**) In negative ion mode: (**F**) Principal component analysis; (**G**) OPLS-DA score plot of Con compared with Mod group; (**H**) OPLS-DA verification diagram; (**I**) OPLS-DA score plot of Mod compared with DHS-H group; (**J**) OPLS-DA verification diagram; (*n* = 7–8).

The preprocessed ileal mucus metabolomics data were imported into the Metaboanalyst 6.0 website for PCA and OPLS-DA analysis. As shown in Figure 6A,F, QC samples showed aggregated distribution in both positive and negative ion modes, which confirmed the stability of the instrument method. In the PCA and OPLS-DA score plots, the Con, Mod, and DHS-H groups showed clear separation in both ion modes (Figure 6A,B,D,F,G,I). This separation indicates that the metabolic profile of ileal mucus was significantly altered in the Mod group compared with the Con group, and that DHS-H treatment shifted the profile away from the Mod group. Specifically, in the OPLS-DA models comparing Con vs. Mod, the R2Y values (positive ion: 0.891; negative ion: 0.795) and Q2 values (positive: 0.638; negative: 0.557) were both high, and the 200-permutation tests (Figure 6C,E) confirmed that the models were not overfit. These results showed that there was a significant difference between the two groups, indicating that SD intervention led to metabolic disorders in ileal mucus. Similar significant separation was observed in the con vs. DHS-H model, with R2Y of 0.749 (positive) and 0.738 (negative), and Q2 of 0.550 (positive) and 0.603 (negative), respectively (Figure 6H,J). These parameters indicate that the metabolic profile of the DHS-H group is not exactly the same as that of the Con group but is significantly different from the Mod group. This indicates that DHS-H treatment partially reversed the metabolic disorders caused by the modeling intervention. In general, multivariate analysis showed that the composition of ileal mucus metabolites in the Mod group changed significantly, and DHS-H administration improved this change, which means that DHS has a potential protective effect on ileal mucus metabolism.

### 3.6. Identification of Differential Metabolites in Ileal Mucus

**Figure 7:**
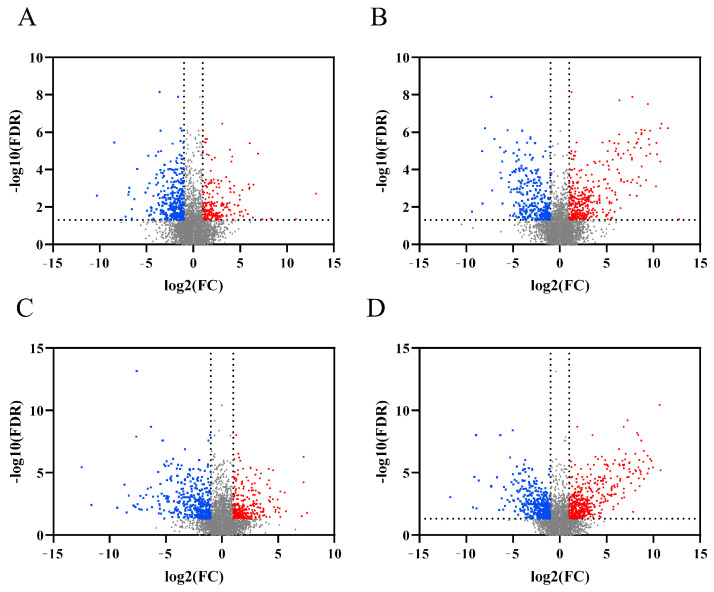
Multiple changes in metabolites are displayed with volcano plots (**A**,**B**). In positive ion mode: (**A**) comparison between the Con group and Mod group; (**B**) comparison between the Mod group and DHS-H group. (**C**,**D**) In negative ion mode: (**C**) Con group and Mod group were compared; (**D**) Mod group and DHS-H group were compared.

The log value of Fold Change (FC) is used as the abscissa, and the -log value of false discovery rate (FDR) is used as the ordinate to draw the volcano plot, as shown in Figure 7. The screened differential metabolites were imported into the METLIN database of MPP15.0 MPP software to match, and the names or molecular formulas of differential metabolites were determined by the information in HMDB and KEGG. A total of 73 differential metabolites were identified in the Con and Mod groups, of which 33 differential metabolites were significantly up-regulated (FDR < 0.05). 40 differential metabolites were down-regulated (FDR < 0.05); a total of 146 differential metabolites were identified in the Mod and DHS-H groups, of which 81 differential metabolites were significantly up-regulated (FDR < 0.05), and 65 differential metabolites were significantly down-regulated (FDR< 0.05).

As shown in Table 1, compared with the sleep deprivation model group, administration of DHS significantly altered the abundance of 10 differential metabolites in the ileal mucus. Specifically, seven metabolites, including testosterone glucuronide, prasterone sulfate, L-urobilinogen, Bilirubin glucuronoside, Bilirubin diglucuronide, 5β-Cyprinol sulfate, and 2-Methoxyestrone 3-glucuronide, were significantly upregulated in the DHS-treated group, while three metabolites, including sulfoglycolithocholate, mesobilirubinogen, and chenodeoxycholylglycine, were significantly downregulated. Two exogenous compounds (triflumuron, tiapamil) were identified as environmental contaminants from bedding and feeding conditions. They were excluded from KEGG pathway analysis to ensure the reliability of endogenous metabolite interpretation.

### 3.7. Ileal Mucus Metabolic Pathway Analysis and Mechanism Verification

**Figure 8:**
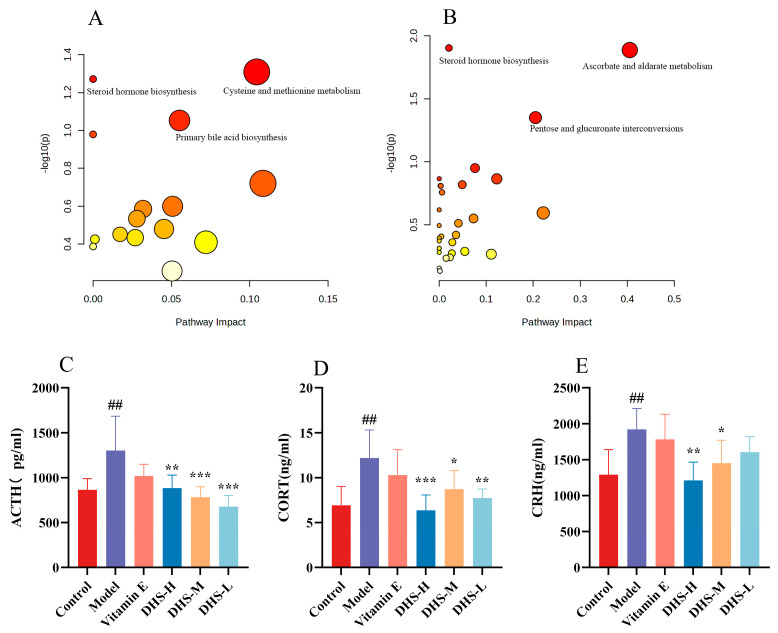
Ileal mucus pathway enrichment analysis and HPA axis verification. (**A**) Comparison of the Con group and Mod group; (**B**) comparison between the Mod group and DHS-H group; (**C**) ACTH content; (**D**) CORT content; (**E**) CRH content (mean ± SD, *n* = 6). Compared with Con group #, *p* < 0.05; ##, *p* < 0.01; ###, *p* < 0.001; compared with Mod group, *, *p* < 0.05; **, *p* < 0.01; ***, *p* < 0.001.

The MetaboAnalyst 6.0 platform was used to analyze the KEGG metabolic pathway of the differential metabolites identified in the ileal mucus of each group. The metabolic pathway screening criteria in the pathway analysis results were set to *p* < 0.1 and Impact ≥ 0.0. As shown in Figure 8. In the comparison between the Con group and the Mod group, three metabolic pathways were enriched, namely Cysteine and methionine metabolism, Steroid hormone biosynthesis, and Primary bile acid biosynthesis. Compared with the Mod group, the DHS-H group was enriched in three metabolic pathways, which were Steroid hormone biosynthesis, Ascorbate and aldarate metabolism, and Pentose and glucuronate interconversions. Among them, the common enrichment pathway in the Con group, Mod group, and DHS-H group was Steroid hormone biosynthesis.

The steroid hormone biosynthesis pathway was enriched by metabolomic analysis of ileal mucus, and this pathway serves as the core downstream effector pathway regulated by the hypothalamic–pituitary–adrenal (HPA) axis. Meanwhile, sleep deprivation, as a typical stressor, can trigger abnormal activation of the HPA axis, thereby modulating metabolic flux and metabolite profiles within this pathway [22]. Corticotropin-releasing hormone (CRH), adrenocorticotropic hormone (ACTH), and cortisol (CORT) are the key components of the HPA axis. As shown in Figure 8, ELISA detection of ACTH, CORT, and CRH in rat serum showed that compared with the Con group, the content of three hormones in the Mod group was significantly increased, and after DHS intervention, the content of three hormones was significantly reduced.

## 4. Discussion

With the accelerating pace of modern life and intensifying social competition, insomnia has become a common and frequently occurring disorder, with its incidence showing a rising trend year by year [23]. Epidemiological data indicate that approximately 10% of adults suffer from insomnia, while 20% experience occasional insomnia symptoms [24]. Insufficient and low-quality sleep can lead to premature death in model organisms, including dogs, rabbits, rats, and fruit flies [25,26]. SD is a known disruptor of circadian rhythms, and disturbances in both sleep quality and rhythm can affect gastrointestinal function [27]. Modern medical research suggests that gut microbial metabolites can influence the expression of central and hepatic clock genes and sleep duration, as well as regulate body composition via circadian transcription factors [28]. Concurrently, bile acid secretion and the enteric nervous system within intestinal tissues are also regulated by circadian rhythms [29,30]. Sleep insufficiency can induce intestinal oxidative stress, generating excessive ROS. MDA is a key product of lipid peroxidation -induced injury, and its increased level is positively correlated with the degree of oxidative damage in the body [31]. GSH is an important endogenous antioxidant involved in scavenging reactive oxygen species and maintaining intracellular redox balance [32]. SOD is an enzyme that catalyzes the dismutation of superoxide anion radicals into H_2_O_2_ and O_2_, serving as a crucial component of the body’s antioxidant defense system [33]. These free radicals can damage lipids, proteins, and DNA, thereby leading to inflammation and chronic diseases [7,34,35]. Intestinal mucus is the first line of defense in intestinal innate immunity. Through its highly glycosylated MUC2 mucin, it provides a key carbon source and habitat for the symbiotic microbiota [36]. Meanwhile, its dense inner layer effectively isolates bacteria from the mucosal epithelium, maintains physical separation and metabolic interaction between host and microorganisms, and its disruption is directly associated with the occurrence of various intestinal inflammatory diseases [37,38,39].

DHS contains a variety of chemical components, such as polysaccharides, amino acids, benzyl compounds, and flavonoids [40]. Clinical studies on DHS have demonstrated its pharmacological effects, including anti-inflammatory, anti-aging, lipid-lowering, hepatoprotective, immunomodulatory, anti-tumor, and antioxidant activities [13,41]. The primary active component of DHS, namely DHS polysaccharides, can regulate type 2 diabetes by improving insulin sensitivity and modulating hepatic glucose metabolism [42]. Current research on the therapeutic effects of DHS on tissue diseases primarily focuses on liver injury and gastrointestinal protection, highlighting its ability to enhance the biochemical, physical, and immune barrier functions of the gastrointestinal tract [43]. Studies have shown that DHS exerts a beneficial effect on the disease states associated with atrophic gastritis, hyperlipidemia, and ulcerative colitis [44,45,46]. This study investigates the reparative effects of DHS on ileal mucosal injury induced by SD in rats, as well as its impact on changes in the properties and composition of intestinal mucus. It further explores the protective role of DHS on the intestinal mucosal barrier.

In this study, DHS effectively ameliorated ileal injury induced by SD, corrected oxidative stress (manifested by increased MDA, and decreased SOD and GSH), and alleviated the dysregulation of inflammatory cytokine secretion (elevated IL-6 and TNF-α, reduced IL-10). Simultaneously, DHS restored the expression of tight junction proteins (E-cadherin and Occludin), indicating its role in enhancing intestinal barrier integrity. SD altered the pH, secretion rate, and composition of ileal mucus. The decreased pH of ileal mucus after DHS treatment reflects a restoration toward normal physiological conditions, which is conducive to optimal mucus viscosity and barrier function. DHS alleviated SD-induced ileal injury primarily by regulating steroid hormone biosynthesis pathways. Activation of the steroid hormone biosynthesis pathway is linked to stimulation of the HPA axis, which plays a crucial role in maintaining homeostasis, responding to stress, and regulating metabolism, immune function, and emotional behavior. DHS significantly reversed the increases in ACTH, CORT, and CRH in rats induced by SD.

This study is the first to link SD-induced ileal dysfunction with alterations in mucus metabolism and to demonstrate the regulatory role of DHS. However, this study only performed ileal mucus metabolomics, and the causal relationship between mucus metabolites and tissue barrier repair remains to be validated. In addition, although the key pathway has been identified, as only the water extract of DHS was used, the main active components (such as polysaccharides, alkaloids, and flavonoids) in DHS require further isolation and identification. Meanwhile, the regulatory network linking steroid hormone biosynthesis and the HPA axis requires further mechanistic study.

## 5. Conclusions

This study confirmed the protective effect of DHS on ileal mucosal injury in sleep-deprived rats by integrating pharmacological and metabolomic approaches. DHS intervention effectively restored ileal tissue structure, alleviated oxidative stress, improved systemic inflammation, and up-regulated the expression of tight junction proteins, thereby enhancing the intestinal physical barrier. Non-targeted metabolomics analysis showed that the therapeutic effect of DHS was primarily mediated by the regulation of specific metabolic pathways, particularly steroid hormone biosynthesis. These pathways are intrinsically linked to mucosal antioxidant defense and detoxification processes. Furthermore, DHS may reduce SD-induced ileal damage by attenuating the abnormal activation of the HPA axis within the steroid hormone pathway. In this study, the protective effect and mechanism of DHS on ileal injury in sleep-deprived rats were investigated through the integration of traditional pharmacodynamics and non-targeted metabolomics.

## Figures and Tables

**Table 1:** Effects of DHS on ileal mucus biomarkers.

Compound	HMDB	KEGG	Formula	Mass	+/−	Trend	
						Con VS Mod	Mod VS DHS-H
Testosterone glucuronide	HMDB0003193	C11134	C_25_H_36_O_8_	464.24	−	↓ ^#^	↑ *
Sulfoglycolithocholate	HMDB0002639	C11301	C_26_H_42_NO_7_S^-^	512.27	−	↑ ^#^	↓ *
Prasterone sulfate	HMDB0001032	C04555	C_19_H_28_O_5_S	368.17	−	↓ ^#^	↑ *
Mesobilirubinogen	HMDB0001898	C05790	C_33_H_44_N_4_O_6_	592.33	−	↑ ^#^	↓ *
L-urobilinogen	HMDB0004157	C05789	C_33_H_48_N_4_O_6_	596.36	+	↓ ^#^	↑ *
Chenodeoxycholylglycine	HMDB0000637	C05466	C_26_H_43_NO_5_	449.31	−	↑ ^#^	↓ *
Bilirubin-glucuronoside	HMDB0060169	C03374	C_39_H_44_N_4_O_12_	760.30	−	↓ ^#^	↑ *
Bilirubin diglucuronide	HMDB0003325	C05787	C_45_H_52_N_4_O_18_	936.33	+/−	↓ ^#^	↑ *
5β-Cyprinol sulfate	HMDB0006888	C05468	C_27_H_48_O_8_S	532.31	−	↓ ^#^	↑ *
2-Methoxyestrone 3-glucuronide	HMDB0004482	C11132	C_25_H_32_O_9_	476.20	+	↓ ^#^	↑ *

Note: Compared with the normal group and the model group, # *p* < 0.05; model group compared with DHS-H group, * *p* < 0.05.

## Data Availability

Data are contained within the article.
